# More, smaller bacteria in response to ocean's warming?

**DOI:** 10.1098/rspb.2015.0371

**Published:** 2015-07-07

**Authors:** Xosé Anxelu G. Morán, Laura Alonso-Sáez, Enrique Nogueira, Hugh W. Ducklow, Natalia González, Ángel López-Urrutia, Laura Díaz-Pérez, Alejandra Calvo-Díaz, Nestor Arandia-Gorostidi, Tamara M. Huete-Stauffer

**Affiliations:** 1 Red Sea Research Center, Division of Biological and Environmental Sciences and Engineering, King Abdullah University of Science and Technology, Thuwal 23955–6900, Saudi Arabia; 2 Instituto Español de Oceanografía, Centro Oceanográfico de Xixón, Xixón, Asturies 33212, Spain; 3 Marine Research Division, AZTI Tecnalia, Sukarrieta, Bizkaia 48395, Spain; 4 Lamont-Doherty Earth Observatory, Columbia University, Palisades, NY 10964, USA; 5 Departamento de Biología y Geología, Física y Química Inorgánica, Universidad Rey Juan Carlos, Móstoles, Madrid 28933, Spain

**Keywords:** bacterioplankton, time series, temperature–size relationships, global warming, long-term trends, Atlantic Ocean

## Abstract

Heterotrophic bacteria play a major role in organic matter cycling in the ocean. Although the high abundances and relatively fast growth rates of coastal surface bacterioplankton make them suitable sentinels of global change, past analyses have largely overlooked this functional group. Here, time series analysis of a decade of monthly observations in temperate Atlantic coastal waters revealed strong seasonal patterns in the abundance, size and biomass of the ubiquitous flow-cytometric groups of low (LNA) and high nucleic acid (HNA) content bacteria. Over this relatively short period, we also found that bacterioplankton cells were significantly smaller, a trend that is consistent with the hypothesized temperature-driven decrease in body size. Although decadal cell shrinking was observed for both groups, it was only LNA cells that were strongly coherent, with ecological theories linking temperature, abundance and individual size on both the seasonal and interannual scale. We explain this finding because, relative to their HNA counterparts, marine LNA bacteria are less diverse, dominated by members of the SAR11 clade. Temperature manipulation experiments in 2012 confirmed a direct effect of warming on bacterial size. Concurrent with rising temperatures in spring, significant decadal trends of increasing standing stocks (3% per year) accompanied by decreasing mean cell size (−1% per year) suggest a major shift in community structure, with a larger contribution of LNA bacteria to total biomass. The increasing prevalence of these typically oligotrophic taxa may severely impact marine food webs and carbon fluxes by an overall decrease in the efficiency of the biological pump.

## 1. Introduction

Climate change is significantly affecting the oceans. Either directly or indirectly [1], the effects of warmer temperatures on marine biota are multiple, but most reports either tackle poleward displacement of lower-latitude species [2] or changes in physiological properties resulting in ecosystem rearrangements [3–5]. Recently, changes in various components of marine food webs, from phytoplankton to mammals [6–8], have been documented, but few reports to date [4] have included the smallest life forms, in spite of their overwhelming importance for standing stocks and biogeochemical cycles [9]. Microbial long-term observations [10] are strongly needed to complement data previously available only for larger groups (phytoplankton and zooplankton).

Heterotrophic prokaryotes dominate the ocean's living biomass [11], mostly comprising Bacteria rather than Archaea in upper layers [12]. In flow cytometric analyses, bacterioplankton cluster into two groups of cells with different nucleic acid content [13,14]. These low (LNA) and high nucleic acid (HNA) groups match bimodal distributions of bacterial genome size [15]. Following universal size–genome relationships [16], HNA bacteria are generally bigger than their LNA counterparts [13,17]. Their ecological significance is still debated, but the emerging consensus is that they represent different lineages [18,19].

Biomass is the combination of abundance and individual size. Although relationships between abundance, body size and temperature are complex [20], two general ecological principles apply: (i) higher abundance is associated with smaller size [21], referred to hereinafter as the abundance–size rule (ASR); and (ii) higher ambient temperature results in smaller individuals according to the temperature–size rule (TSR) [22], little studied in unicellular organisms [23]. While the underlying mechanism for the ASR is clear (constant biomass at a given level of resources implies that if there are more organisms these should be smaller), the causes for the TSR are more elusive, with several alternative hypotheses (e.g. [24]). We recently assessed the validity of these two rules to explain changes in phytoplankton size-structure using data collected across the North Atlantic [25]. Similar to that study, in which changes in the overall phytoplankton size community composition rather than intraspecific changes were addressed, we provide here one of the first attempts to detect shifts in the composition and size of planktonic heterotrophic bacteria. Although claims have been made that body size will universally decrease as a consequence of climate change [26,27], studies targeting the smallest life forms are lacking. Here, we explored the seasonal and interannual patterns of LNA and HNA bacteria, focusing on their temperature responses through the ASR and TSR, in a 10-year oceanographic dataset from the southern Bay of Biscay continental shelf, in order to shed light on future directions of change of microbial plankton.

## 2. Material and methods

### (a) Environmental variables

The study site on the Southern Bay of Biscay continental shelf (43.67° N, 5.58° W, bottom depth 110 m) off Xixón, Spain, has been monitored monthly since 2001 as part of a time series programme. Physico-chemical and environmental characterization is detailed in [17]. The depth of the upper mixed layer was determined as that where more than or equal to 0.05 kg m^−3^ density increase over 5 m was first observed. A stratification index was calculated as the per-metre difference in temperature between the surface and 75 m.

### (b) Bacterioplankton abundance, cell size and biomass

Bacterioplankton samples were taken at 10–25 m intervals from April 2002 to March 2012, and analysed with a FACSCalibur (BD) flow cytometer as described in detail in [17]. Briefly, LNA and HNA cells were distinguished after nucleic acid staining in green fluorescence versus right-angle light scatter (RALS) cytograms (electronic supplementary material, figure S1*a*). Bacterial abundance (cells ml^−1^) was estimated after daily calibrating the flow rate. When present, the high natural fluorescence of *Prochlorococcus* due to photosynthetic pigments prevented overlap with the HNA cluster in red versus green fluorescence cytograms (electronic supplementary material, figure S1b). Cell size (µm^3^) was obtained with an empirical calibration between cell diameter and mean RALS, because of its higher sensitivity [28] compared with forward angle light scatter, assuming spherical shape [17]. This assumption may have introduced biases especially in rods or curved rods such as most SAR11 cells, abundant in our samples (see below). Cell size was converted into biomass using [29]: pg C cell^−1^ = 0.12 × cell size^0.72^. LNA and HNA bacterial biomass (µg C l^−1^) was fundamentally driven by changes in abundance.

### (c) Quantification of SAR11 phylotype in environmental samples

The contribution of the SAR11 clade to total abundance was assessed by catalysed reporter deposition fluorescence *in situ* hybridization (CARDFISH). For CARDFISH analysis, 4.5 ml samples were collected monthly in 2012, fixed with 3.7% formaldehyde for 3 h, filtered onto 0.2 µm pore-size polycarbonate filters and frozen until analysis. Hybridization was performed as described in [30] using the probe SAR11–441R targeting the SAR11 cluster [31]. Counterstaining of CARDFISH preparations was done with 4,6-diamidino-2-phenylindole (DAPI) at 50 µg ml^−1^. Cells were counted with a Leica DM 5500 B epifluorescence microscope and pictures were taken with a Leica DFC 360FX monochromatic camera. The abundance and size of SAR11-positive cells were calculated using AcmeTool2 image analysis software [32] and the algorithm by Massana *et al*. [33], which yielded length and width of each cell subsequently used for estimating biovolume.

### (d) Time series analysis

Time series analysis was conducted with bacterial and environmental properties averaged for the upper mixed layer, with minima usually observed in summer (15 ± 1 m s.e.) and maxima in winter (58 ± 8 m). We used an additive decomposition time series model [34] to detect seasonal and long-term components. The variability of the time series (*X*) was expressed as
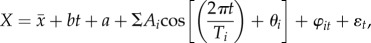
where the time series was represented by the climatological mean 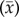, the linear trend with the slope (*b*) and intercept (*a*) of the linear regression with time (*t*), the periodic components amplitude (*A_i_*), period (*T_i_*) and phase (*θ*_*i*_), the autocorrelation coefficients between consecutive values (*φ*_*it*_) and the unexplained residuals (*ɛ*_*t*_). We used the Fisher *G*-test for assessing the significance of periodic components. Correlation analysis between variables was subsequently made with the pre-whitened residuals (i.e. once we had filtered out the seasonal and long-term components and adjusted for autocorrelation). Decadal trends were also computed with annual averages.

### (e) Temperature–size experiments

In 2012, we performed 12 monthly incubations with surface samples from the same site aimed at determining the bacterial response to temperature, both in the presence of the whole microbial community and with bacteria only, after pre-filtering the sample through 0.8 µm pore-size filters. Triplicate bottles (2 l) were incubated at three temperatures (*in situ*, −3°C and +3°C), and samples for estimating LNA and HNA abundance and cellular properties were taken twice per day for 5–10 days. Although seasonally very similar, we will include here only the results of the filtered incubations to exclude trophic interactions with other components. Healthy bacterial populations were invariably found, which started to grow shortly after confinement. HNA cells grew always faster than their LNA counterparts. The slopes of model I linear regressions of mean cell size versus experimental temperature were used as estimates of the monthly responses of LNA and HNA bacterial sizes to warming (µm^3^ °C^−1^).

## 3. Results

Overall, bacterial abundance ranged from 1.2 to 31 × 10^5^ cells ml^−1^, with HNA cells being slightly more abundant (mean 54 ± 1% s.e.). The vertical decrease in abundance was more marked from 40 m downwards (electronic supplementary material, figure S2a). Mean bacterial cell size ranged from 0.032 to 0.115 µm^3^ (electronic supplementary material, figure S2b), with HNA cells significantly higher than LNA (0.056 and 0.050 µm^3,^ respectively, paired *t*-test, *p* < 0.001, *n* = 114). Mean cell sizes reached a minimum at 40 m and then increased slightly down to the seafloor. Larger sizes were significantly correlated with higher nucleic acid content (electronic supplementary material, figure S3).

### (a) Seasonal patterns

**Figure 1.**
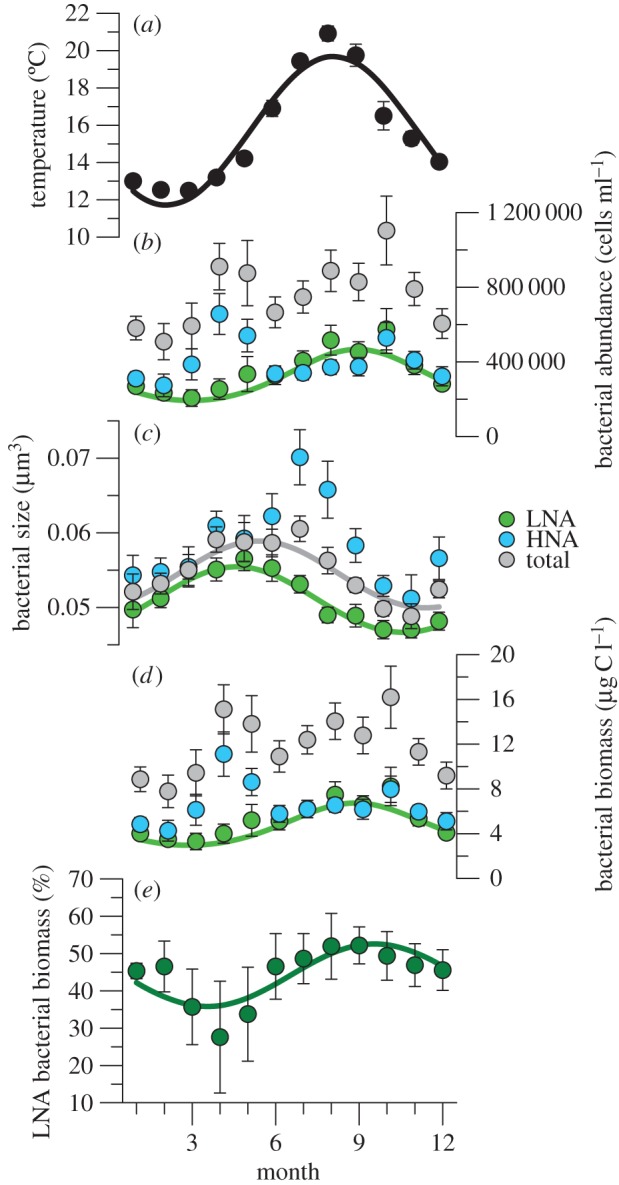
Seasonal variations of temperature and bacterioplankton. Monthly mean ± s.e. values of (*a*) temperature, and (*b*–*d*) total, LNA and HNA (*b*) bacterial abundance, (*c*) bacterial size, (*d*) bacterial biomass and (*e*) per cent contribution of LNA bacteria to total biomass in the upper mixed layer of the study site for the April 2002–March 2012 period. Fitted curves represent statistically significant seasonality detailed in table 1.

Temperature displayed a marked seasonality (figure 1*a*), with the 12-month periodic component explaining 80% of total variance (table 1). HNA bacteria had no persistent cyclical components, whereas LNA bacteria showed a statistically significant annual cycle explaining 25% of total variance (figure 1*b* and table 1). The spring and autumn peaks in total bacterial abundance (approx. 10^6^ cells ml^−1^; figure 1*b*) and biomass (approx. 16 µg C l^−1^; figure 1*d*) were characterized by a different relative contribution of both groups, resulting in a strong seasonality of the percentage contribution of LNA cells to bacterial biomass (figure 1*e*). Earlier work had already shown a very strong seasonal signal of the percentage of HNA cell abundance, with approximately 40% minima in summer and maxima more than 80% around April [17], which is definitely confirmed with this larger dataset. However, little was known about the temporal variability in HNA and LNA bacterial cell size, and no prior attempt had been made at relating their seasonal and long-term patterns to temperature. Bacterial cell size had significant seasonal components for LNA and total bacteria (figure 1*c* and table 1), with maxima and minima lagged by roughly one month because of the marked summer peak in HNA cell size. For LNA bacteria, seasonal patterns of abundance and size were roughly opposite, and pooled LNA cell abundance and size were negatively correlated (*r* = −0.33, *p* < 0.001, *n* = 114). LNA cell size was also negatively correlated with temperature (*r* = −0.19, *p* = 0.044, *n* = 114). Other variables concurrently measured and potentially relevant for bacteria include total chlorophyll (size-fractionated also since 2003), inorganic nutrient concentrations and stratification index. Briefly, marked stratification from June to October was accompanied by strong nutrient limitation, resulting in low chlorophyll and picophytoplankton dominance. Chlorophyll usually peaked around March–May, with greater contributions of the larger size-fractions. The variance decomposition of these ancillary variables is shown in electronic supplementary material, table S1.

**Table 1.** Variance decomposition of the upper mixed layer bacterial times series (April 2002–March 2012) at the study site for total, LNA and HNA cells, and the percentage contribution of LNA cells to total biomass (%LNA biomass). Abundance (cells ml^−1^), size (µm^3^) and biomass (µg C l^−1^) variables were log_10_ transformed. Only significant (*p* < 0.05) variance components are shown, indicating the fraction of total variance accounted for (%var). Slope (*b*), intercept (*a*), period in months (*T*), amplitude (*A*), phase in radians (*θ*), time when maximum value occurs in months (*T*_max_), autocorrelation coefficient (*Φ*). Lag in months.

		linear trend			periodic components					autocorrelation			total
variable	mean	*b*	*a*	%var	*T*	*A*	*θ*	*T*_max_	%var	lag	*Φ*	%var	%var
temperature	15.7	—	—	—	12	3.89	4.26	8.13	80.43	1	0.34	2.28	82.71
total abundance	5.83	—	—	—	—	—	—	—	—	1	0.20	4.32	4.32
LNA abundance	5.47	0.016	−32.50	2.63	12	0.19	4.69	8.95	25.50	—	—	—	28.13
HNA abundance	5.55	—	—	—	—	—	—	—	—	—	—	—	—
total size	−1.27	−0.005	10.53	8.73	12	0.04	2.92	5.58	26.31	—	—	—	35.04
LNA size	−1.29	−0.004	7.25	4.19	12	0.04	2.44	4.67	31.84	—	—	—	36.05
HNA size	−1.24	−0.007	13.17	7.07	—	—	—	—	—	—	—	—	7.07
total biomass	1.02	—	—	—	—	—	—	—	—	1	0.21	4.77	4.77
LNA biomass	0.65	—	—	—	12	0.18	4.56	8.72	23.01	—	—	—	23.01
HNA biomass	0.76	—	—	—	—	—	—	—	—	—	—	—	—
%LNA biomass	44.2	0.59	−1179	2.58	12	8.40	5.00	9.54	31.32	—	—	—	33.90

### (b) Comparison between SAR11 and flow-cytometric groups

Both the abundance and individual size of SAR11 bacteria covaried positively with flow-cytometric LNA cell values but not with those of HNA cells (electronic supplementary material, figure S4*a*–*d*), strongly suggesting that LNA cells were mainly composed of SAR11 bacteria. If all SAR11 cells were in the LNA cluster, as current knowledge supports (see Discussion), their mean annual contribution to LNA bacterial abundance would be 74% ± 16%. With regard to size, although strongly correlated (electronic supplementary material, figure S4*c*), the mean 28% lower size of LNA compared with that of SAR11 cells should not be attributed, in our opinion, to the presence of smaller bacteria, but to the different methods used (flow cytometry versus microsocopic image analysis).

### (c) Long-term trends

**Figure 2.**
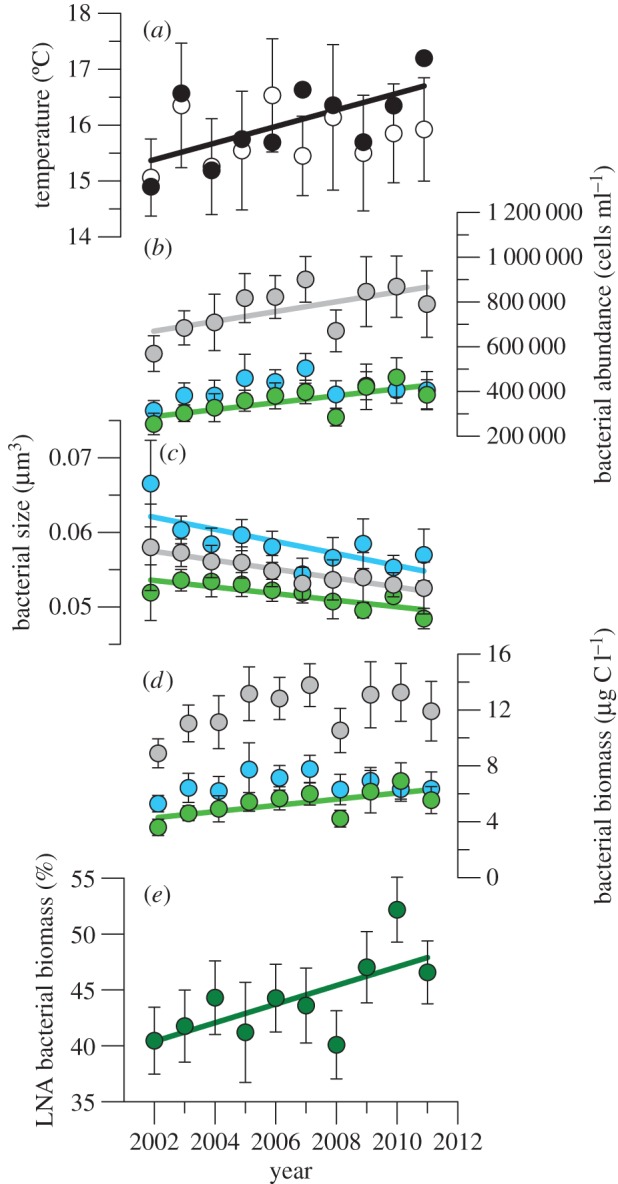
Long-term trends of temperature and bacterioplankton. Annual (April–March) mean ± s.e. values of (*a*) temperature, and (*b*–*d*) total, LNA and HNA (*b*) bacterial abundance, (*c*) bacterial size, (*d*) bacterial biomass and (*e*) percentage contribution of LNA bacteria to total biomass in the upper mixed layer of the study site. Filled symbols in (*a*) represent average temperatures for the April–July period. Error bars for this period are not shown but were on average 48% higher than the annual mean s.e. values. Fitted continuous lines represent significant trends detailed in electronic supplementary material, table S1.

In addition to the seasonal patterns, we also identified conspicuous decadal trends (table 1). Figure 2 shows them as linear regressions of annual mean values (electronic supplementary material, table S2). In two cases (total bacterial abundance and LNA bacterial biomass), annual mean values increased significantly with time although the variance decomposition of the whole time series failed to find linear trends. The increase in annual mean temperatures was not significant, partly because of the large weight of outliers in low-frequency monitoring. Examining monthly temperature changes (data not shown), the only consistent period of warming extended from April through July, with October and November presenting cooling trends. Once in September and twice in October, coastal upwelling caused notable cooling. Consequently, we considered also mean temperatures for the April–July period, coincident with the steepest yearly increase (figure 1*a*). A significant decadal warming of approximately 1.5°C became apparent for April–July mean temperatures (figure 2*a*), while mean values for the rest of the year showed a non-significant decrease. The large differences between the annual and spring–early summer temperatures in 2007 and 2011 (figure 2*a*) were largely due to the above-mentioned strong upwelling events, decreasing almost 4°C the expected monthly values. The abundance of LNA cells increased significantly during the decade (figure 2*b*), adding approximately 3% to the variance explained by seasonality (table 1). Although HNA cells did not show persistent cyclical components, the negative decadal trend in HNA cell size explained a larger percentage of variance than declining LNA cell size, also reflected in steeper slopes (table 1; electronic supplementary material, table S2). Total bacterioplankton cell size decreased consistently from 2002 to 2012 (equivalent to approx. 1% shrinking per year) as a combined consequence of the sustained decrease in the size of both groups (figure 2*c*) and the positive trend in the abundance of the smaller LNA bacteria, which also increased significantly in biomass when annual values were considered (figure 2*d*; electronic supplementary material, table S2). The mean contribution of LNA cells to total bacterial biomass significantly increased from 40 to 47% between 2002 and 2012 (figure 2*e* and table 1; electronic supplementary material, table S2).

### (d) Residual analysis

The correlations between pre-whitened residuals of bacterial properties and selected environmental variables are shown in table 2. Temperature residuals were significantly correlated with both LNA and HNA cell size residuals, but negatively in the former and positively in the latter. Although the relationship between temperature and the contribution of LNA bacteria to total biomass was not significant, a significant correlation was found with LNA cells contribution to total numbers (*r* = 0.21, *p* = 0.021, *n* = 120). The residuals of the contribution of LNA bacteria to total biomass were also positively correlated with those of stratification index and nitrate concentrations, and negatively with total chlorophyll. The latter correlation became more negative with the absolute and relative concentrations, of chlorophyll in the microplankton size class. Total and size-fractionated chlorophyll residuals were also variably associated with the residuals of LNA and HNA cells, summarized by a negative effect of total chlorophyll on HNA cell size (and positive on HNA abundance) and a positive effect on LNA cell size, more marked with large than small phytoplankton.

**Table 2.** Pearson correlation coefficients between the pre-whitened residuals of heterotrophic bacterioplankton and selected environmental variables. SI, stratification index; NO_3_, nitrate concentration; Chl, total chlorophyll; pChl, chlorophyll smaller than 2 µm; nChl, chlorophyll 2–20 µm; mChl, chlorophyll larger than 20 µm; %pChl, %nChl and %mChl, percentage contribution to Chl of pChl, nChl and mChl, respectively. *n* = 114.

	total abund	LNA abund	HNA abund	total size	LNA size	HNA size	total biomass	LNA biomass	HNA biomass	%LNA biomass
temperature	—	—	—	—	−0.19*	0.26**	—	—	—	—
SI	—	—	−0.23**	0.19*	—	0.34***				0.23*
NO_3_	−0.23*	—	−0.31**	—	—	—	−0.22*	—	−0.30**	0.31**
Chl	—	—	0.18*	—	0.19*	−0.25**	—	—	—	−0.19*
pChl	—	—	—	—	—	—	—	—	—	—
nChl	0.22*	—	—	—	—	—	0.23*	—	0.18*	—
mChl	—	—	0.25**	—	0.27**	—	—	—	0.23*	−0.27**
%pChl	—	—	—	−0.19*	−0.23*	—	−0.19*	—	—	—
%nCHl	—	—	—	—	—	—	—	—	—	0.22*
%mChl	—	—	0.19*	—	0.20*	—	—	—	—	−0.27**

***p < 0.001; **p < 0.01, *p < 0.05.

### (e) Temperature–size experiments

**Figure 3.**
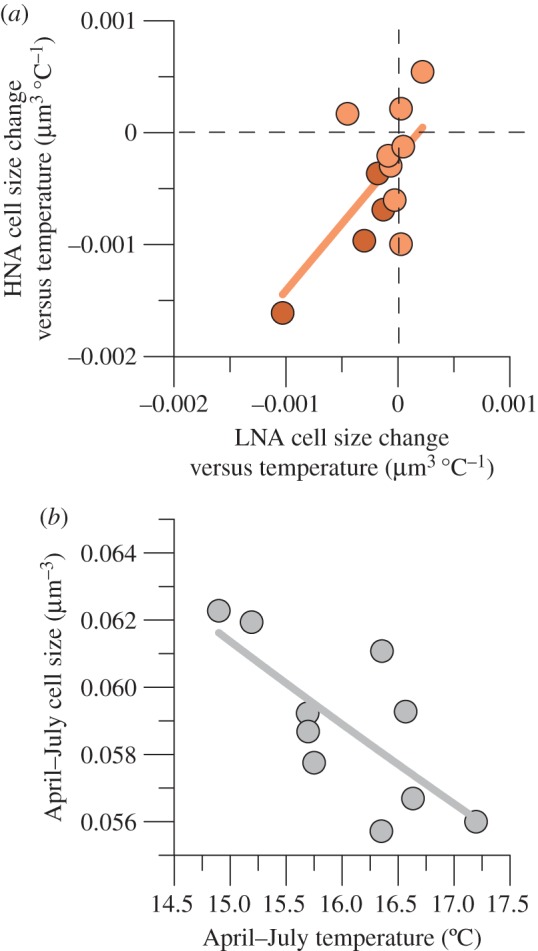
(*a*) Comparison between cell size change versus temperature (ccc-temp) for LNA and HNA bacteria in experimental incubations with surface samples taken in 2012. Darker symbols represent results from April through July. Fitted line: HNAccc-temp = −0.0002 + 1.19 LNAccc-temp, *r*^2^ = 0.41, *p* = 0.024, *n* = 12. Dashed lines represent no change (0 slope of the linear regression, more details given in the text). (*b*) Relationship between mean values of bacterial cell size and temperature for the period extending from April through July of the 10 years of available data. Fitted line: April–July cell size + 0.098 − 0.024 April–July temperature, *r*^2^ = 0.52, *p* = 0.017, *n* = 10.

The 6°C gradient in experimental temperature generally resulted in smaller bacterial cell sizes, both for LNA (eight out of 12 experiments) and HNA cells (nine experiments), as indicated by negative slopes in the cell size versus temperature linear regressions. The respective monthly cell size versus temperature changes were significantly correlated for both groups (figure 3*a*), with an average 2.2-fold higher decrease in HNA than in LNA cells. Both groups showed highly coherent cell size decreases with temperature from March to September (data not shown), particularly marked for spring–early summer (*n* = 4), with mean values of −0.00041 and −0.00091 µm^3^°C^−1^ in LNA and HNA bacteria, respectively. Figure 3*b* shows that mean values of bacterial cell size and temperature for the April–July period of the time series were also significantly and negatively correlated.

## 4. Discussion

The decadal time series analysis of the LNA and HNA bacterial groups indicates that seasonal changes in the abundance and cell size of the LNA group were coherent with both the TSR and the ASR (i.e. more abundant and smaller LNA bacteria were found during the warmest months). Although the ASR is based on energetic equilibrium, which probably does not hold in a seasonally varying environment, the analysis of the entire LNA bacteria dataset is consistent with the general ASR prediction that the higher the abundance, the smaller the size of an organism [21]. Based on the good agreement between the size of LNA and SAR11 bacteria, the latter derived from the more precise image analysis of DAPI-stained samples (electronic supplementary material, figure S4c), our assumption of spherical form in flow cytometry data probably had a minor effect on biovolume estimates, at least regarding the relative differences between groups and over time that were the object of this study.

We explain the coherent behaviour of the LNA cells by their lower diversity (electronic supplementary material, figure S5), probably dominated by a single alphaproteobacterial clade, SAR11 (electronic supplementary material, figure S4), as previously found [18,31,35]. Genome sizes of cultured SAR11 strains would place them neatly in the LNA rather than the HNA cluster [36,37]. In a large cross-Atlantic survey, LNA populations were invariably dominated by SAR11, while HNA cells were phylogenetically diverse, including members of Bacteroidetes, Gammaproteobacteria and other Alphaproteobacteria lineages [18]. Numerous ecotypes with different metabolic functions and temperature dependences are grouped under the SAR11 lineage [38,39], which could help explain the slightly higher presence of SAR11 in winter in the Pacific [40], contrary to common observations of summer abundance maxima [38,41] (this study). At our site, two SAR11 ecotypes were found among the top 10 most abundant bacterial OTUs (plus eight more in the top 100) in a 3.5-year survey [42]. Their biogeochemical roles and the existence of differing long-term trends exceed the scope of this study, but are part of ongoing efforts. On the contrary, the higher diversity (electronic supplementary material, figure S5) and marked species succession within HNA cells [18,19] may be related to the absence of seasonality and of significant correlation between abundance and size. Lack of bacterial time series through sufficiently extended periods precludes assessing the generality of our observations. Yet there is evidence of seasonally recurring patterns in phylogenetic community composition in temperate systems [43,44], including our own site [42]. These cyclical changes can also be discerned in the much coarser flow cytometric classification (figure 1).

While 10 years were too short to find sustained warming of the upper mixed layer, in recent decades, the oceans have warmed up at an unprecedented pace (e.g. [45]). Extended analysis (electronic supplementary material, figure S6) and longer records just 50 km eastwards show that the southern Bay of Biscay is no exception, with an approximate 0.05°C yr^−1^ increase [46] comparable with other latitudes. This increase has not been homogeneous and our results indicate that significant spring–early summer warming (figure 4*a*) was accompanied by non-significant cooling for the rest of the year. Seasonally uneven warming is widespread, with reports for our study area consistently agreeing on the preponderance of higher summer temperatures over winter values, including the whole northwestern European continental shelf [47], the Iberian Peninsula [48] and the above-mentioned nearby site [49]. Based on monthly snapshots rather than continuous measurements, decadal trends were more compelling for bacterial characteristics than for temperature, probably due to the fact that bacterioplankton integrate environmental forcing over periods longer than days. Nevertheless, the inverse relationship found between spring–early summer mean temperatures and bacterial sizes (figure 3*b*) might be linked to the period of the year in which larger decreases in LNA and HNA cell volume with experimental temperature were observed (figure 3*a*).

We are not aware of previous studies concurrently assessing temporal trends of marine bacterioplankton abundance and cell size. The few reported time series of heterotrophic bacteria are mostly restricted to coastal temperate waters, for which consistent interannual decreases [50] and increases in abundance [51] were associated with parallel changes in phytoplankton standing stocks. Recent increases in heterotrophic bacteria and picophytoplankton in polar environments were explained by enhanced sea ice melting and decreased nutrient supply [52], which can still lead to higher primary production, largely channelled through dissolved organic compounds usable by bacteria [53]. Availability of resources for bacterial uptake could be an alternative hypothesis to temperature explaining the observed trends. Using phytoplankton as a proxy for resource availability, high values should result in high bacterial abundances [54]. This could explain the spring peaks in HNA bacterial abundance (figure 1*b*), positively correlated with chlorophyll (*r* = 0.18, *p* = 0.048, *n* = 114; see also table 2). However, no correlation was found with LNA cells, and neither total chlorophyll nor any size-fraction showed decadal trends (electronic supplementary material, table S1). Recent work concurs that LNA cells are spatio-temporally independent of phytoplankton, at least at short scales [38,55], although a study in the Pacific has found positive correlations of SAR11 with DOC and primary production [40]. LNA/SAR11 cells could also have benefitted from environmental conditions related to high temperature, such as low inorganic nutrients content and high light, foreseen to expand in the future ocean [1,56]. Although environmental variables other than temperature also showed seasonal and long-term trends (electronic supplementary material, table S1), the only ones that changed intra- and interannually coherently with bacterial properties were inorganic nutrient concentrations. However, while the seasonal patterns of NO_3_ and PO_4_ and LNA cell abundance were roughly opposite (cf. table 1; electronic supplementary material, table S1), both nutrients increased rather than decreased with time, but this increment did not translate into larger phytoplankton biomass.

Because of the covariance and linear trends of most variables, correlation analysis of residual variation (table 2) is more suitable to search for mechanistic explanations. The preferential association of HNA cells with phytoplankton (large rather than small) was confirmed by residual analysis. Although nitrate concentration residuals showed correlations of opposite sign with HNA cell abundance and %LNA biomass, it is difficult to see nitrate availability as the direct driver when phosphate residuals were not correlated (data not shown) and without concomitant changes in phytoplankton. The negative correlation between temperature and LNA cell size residuals confirms the compliance with the TSR of LNA bacteria, which also prevailed in abundance in warmer waters. Their positive association also with the water-column thermal gradient (table 2) suggests that indirect effects of global warming such as enhanced stratification [46,47,57] might exacerbate the future prevalence of LNA cells. Altogether, the match between seasonal and decadal trends and residual analysis identify temperature as the candidate for the dominance of increasingly smaller LNA bacteria in these waters. Finer-scale variations within the group (e.g. changes in the relative contribution of SAR11 ecotypes with differing temperature ranges [38,39]) may be behind the observed patterns.

Smaller phytoplankton and zooplankton in response to rising temperatures have been demonstrated both experimentally and through sustained observations [4,25,58,59], but as far as we know this is the first study documenting a systematic long-term decline in bacterioplankton size, thus supporting the view of decreasing body size as a universal ecological response to warming [26,27] alongside changes in phenology [60] and distributional ranges [2]. The decadal trends of declining total, LNA and HNA bacterial sizes (figure 2*c*) are highly relevant, particularly considering that only LNA cells consistently followed the ASR and TSR on the two temporal scales (seasonal and interannual) compared here. Seasonality of HNA cells, bimodal for abundance and unimodal for size (figure 1*b*,*c*), did not preclude a decadal compliance with the TSR. While the seasonal patterns of LNA and HNA bacterial size differed markedly, probably linked in the latter case to the recurrent appearance of large species in spring and summer [42], both groups decreased consistently in size as temperature rose. In the 10 years covered by this study, surface warming was better detected in the period extending from April through July than in the annual average (figure 2*a*).

Bearing in mind that a gradual ocean warming of 0.5°C per decade cannot be directly comparable with a quasi-instantaneous 3°C increase in an incubation, the hypothesis that temperature is the ultimate cause for the marked decrease in bacterial mean size (approx. 1% yr^−1^) is further strengthened by the experimental results with natural samples in which temperature was the only factor manipulated (figure 3*a*). Interestingly, the size of HNA cells tended to decrease more markedly than LNA cells both interannually (figure 2*c*; electronic supplementary material, table S2) and experimentally (figure 3*a*). Applying the 2012 mean spring–early summer decrease of LNA and HNA cell size per °C (figure 3*a*, darker symbols) to the 2002–2011 April–July warming (figure 2*a*) would result in an overall size decrease of 3%. This value is smaller than the observed 10% (figure 2*c*), possibly related to the hugely different temporal scales compared (days versus years). Negative associations between temperature and bacterial size have been documented in large spatial surveys [61] as well as experimentally through reductions in genome size [62], usually strongly correlated with cell volume [16] (electronic supplementary material, figure S3).

Atlantic cross-ocean analysis [25] hints at a rapid increase in the foreseen contribution of cyanobacteria to planktonic communities [63]. Residual variation analysis (table 2) suggests that the future prevalence of small phytoplankton may also enhance LNA bacterial contribution to total stocks. The implications of a future dominance of small autotrophic and heterotrophic bacteria for oceanic food webs and biogeochemistry are multiple. The size structure of planktonic communities affects ecosystem functions such as predator–prey interactions, efficiency of energy transfer across trophic levels and ultimately carbon export [5,64]. Gradual replacement of bacteria by typically oligotrophic taxa such as the SAR11 clade and others included within the LNA cluster [14,18,35] will be likely to alter carbon fluxes and trophic relationships as these bacteria grow slowly [65] and, contrary to HNA cells, appear not to respond simultaneously to changes in phytoplankton primary production [55]. We document here a rapid change in bacterioplankton community composition that probably follows fundamental ecological and metabolic rules. This study adds to evidence from recent and geological times [4,59] to strongly support that, regardless of the trophic level and biological organization, a shift towards lower sizes can be a widespread response of marine organisms to global change. Additional studies are needed to test these associations between warming, bacterial abundance and cell size, but only longer oceanographic time series will confirm the generality of shrinking marine bacteria. The consequences for ecosystem functioning are far-reaching, but can be summarized as a lower transfer efficiency of primary production towards higher trophic levels together with a decrease in the importance of the biological pump for efficient carbon sequestration in the ocean's interior.

## Supplementary Material

Moran et al ESM 210415

## Data accessibility

All data are deposited in Dryad (manuscript Dryad ID: http://dx.doi.org/10.5061/dryad.kh7nt).
