# Supplementary material for: More, smaller bacteria in response to ocean's warming?
Source: Proc Biol Sci. 2015 Jul 7;282(1810):20150371. doi: 10.1098/rspb.2015.0371 (PMC4590472; doi:10.1098/rspb.2015.0371)
Supplement: Moran et al ESM 210415 [file rspb20150371supp1.pdf]

Electronic Supplementary Information (ESM)

More, smaller bacteria in response to ocean's warming?

Xosé Anxelu G. Morán[1,2*], Laura Alonso-Sáez[2,3], Enrique Nogueira[2], Hugh W. Ducklow[4], Natalia González[5], Ángel López-Urrutia[2], Laura Díaz-Pérez[2], Alejandra Calvo-Díaz[2], Nestor Arandia-Gorostidi[2], Tamara M. Huete-Stauffer[2]

[1] Red Sea Research Center, King Abdullah University of Science and Technology Thuwal 23955-6900 Saudi Arabia

[2] Instituto Español de Oceanografía, Centro Oceanográfico de Xixón, Camín de L'Arbeyal, s/n, E-33212 Xixón, Asturies, Spain

[3] AZTI

[4] Lamont-Doherty Earth Observatory, Palisades, NY 10964, USA

[5] Univ. Rey Juan Carlos, Depto. Biología y Geología, 28933 Móstoles, Madrid, Spain

Table S1. Variance decomposition of upper mixed layer-averaged selected environmental variables. Total chlorophyll (Chl) and in 3 size-classes (pChl, <2 µm; nChl, 2-20 µm; mChl, >20 µm): SI, stratification index; Zuml, upper mixed layer depth. Only significant ($p<0.05$) components are shown, indicating the fraction of total variance explained (%var). (b) slope, (a) intercept, (T) period in months, (A) amplitude, (θ) phase in radians, (T_{max}) time when maximum value occurs in months, (Φ) autocorrelation coefficient. Lag in months.

		Linear trend			Periodic components					Autocorrelation			Total
**Variable**	**Mean**	**b**	**a**	**%var**	**T**	**A**	**θ**	T_{max}	**%var**	**Lag**	**Φ**	**%var**	**%var**
SI	0.04	---	---	---	12	0.06	4.09	7.81	81.17	1	0.36	2.52	83.70
Zuml	34.65	---	---	---	12	28.02	0.54	1.03	32.39	---	---	---	32.39
daylength	12.00	---	---	---	12	3.26	3.22	6.15	99.32	---	---	---	99.32
NO_3	0.38	0.022	-44.51	5.94	12	0.30	0.65	1.23	66.10	---	---	---	72.03
NO_2	0.06	0.005	-9.21	4.77	---	---	---	---	---	1	0.31	8.90	13.68
PO_4	0.08	0.005	-9.95	8.41	12	0.05	0.61	1.17	51.98	---	---	---	60.39
SiO_2	0.34	---	---	---	12	0.21	0.69	1.32	52.58	1	0.35	5.72	58.30
Chl	-0.33	---	---	---	---	---	---	---	---	1	0.20	3.89	3.89
pCHl	-0.81	---	---	---	12	0.20	0.05	0.09	22.86	1	0.25	4.71	27.58
nCHl	-0.81	---	---	---	12	0.26	1.58	0.83	29.25	---	---	---	29.25
mCHl	-1.11	---	---	---	---	---	---	---	---	---	---	---	---
%pChl	37.04	---	---	---	12	0.12	5.03	9.61	29.02	---	---	---	29.02
%nCHl	38.51	---	---	---	---	---	---	---	---	---	---	---	---
%mChl	23.08	---	---	---	---	---	---	---	---	---	---	---	---

Table S2. Significant ($p < 0.05$) interannual trends indicated by Model I (ordinary least squares) linear regressions between year and annual (Apr-Mar) mean values of temperature, abundance (cells mL^{-1}), cell size (μm^3) and absolute and relative biomass ($\mu g\ C\ L^{-1}$) of total, LNA and HNA bacteria. Also shown is the trend of annual mean temperatures calculated for a third of the months (from April through July, details given in the main text). All bacterial variables except the percent contribution of LNA cells to total biomass were log-10 transformed. b, slope; a, intercept; r^2, coefficient of determination; p, significance level. --- not significant. Values in brackets represent standard error of estimates.

**Variable**	**b**	**a**	**r^2**	**p**
Temperature	---	---	---	---
Apr-Jul temperature	0.148 (0.063)	15.22 (0.40)	0.40	0.049
Total abundance	0.013 (0.006)	5.80 (0.03)	0.41	0.046
LNA abundance	0.019 (0.007)	5.44 (0.04)	0.52	0.019
HNA abundance	---	---	---	---
Total size	-0.005 (0.001)	-1.24 (0.00)	0.91	<0.001
LNA size	-0.004 (0.001)	-1.27 (0.01)	0.65	0.005
HNA size	-0.006 (0.002)	-1.20 (0.01)	0.53	0.017
Total biomass	---	---	---	---
LNA biomass	0.019 (0.007)	0.61 (0.05)	0.46	0.032
HNA biomass	---	---	---	---
%LNA biomass	0.83 (0.32)	39.6 (1.96)	0.46	0.030

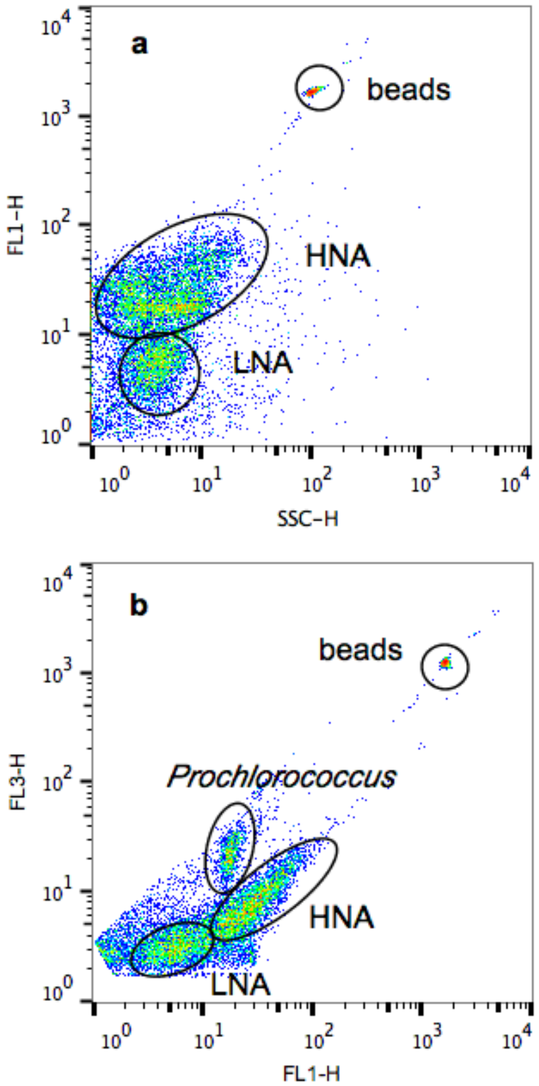

Fig. S1. Example of cytograms from the bacterioplankton time-series (30th October 2012, 20 m depth) showing the two groups of heterotrophic bacteria differentiated according to their relative nucleic acid content after Syto-13 statining, low (LNA) and high (HNA). (a) Y-axis represents green fluorescence (FL1) while the X-axis is right angle light scatter (RALS/SSC), a surrogate of cell size. The HNA cell cluster in this plot include *Prochlorococcus* cyanobacteria. (b) Y-axis represents red fluorescence (FL3) and X-axis green fluorescence. *Prochlorococcus* cells here yield a distinctly higher red fluorescence signal that allows clear differentiation from heterotrophic HNA bacteria. A few *Synechococcus* cells are also visible with higher FL3 signal. Beads are 1 µm in diameter and were used as an internal standard. See the methods section for details.

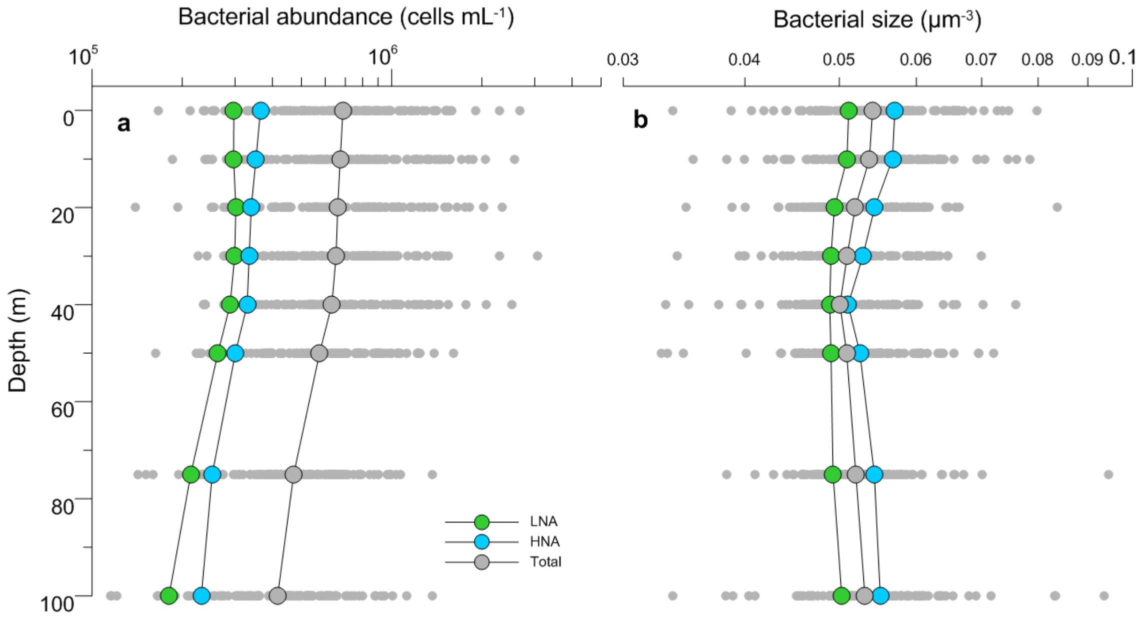

Fig. S2. Vertical distribution of (a) the abundance and (b) the mean cell sizes of LNA, HNA and total bacteria at the study site for the April 2002-March 2012 period. All meaured data are represented by grey dots and mean values per depth as coloured dots.

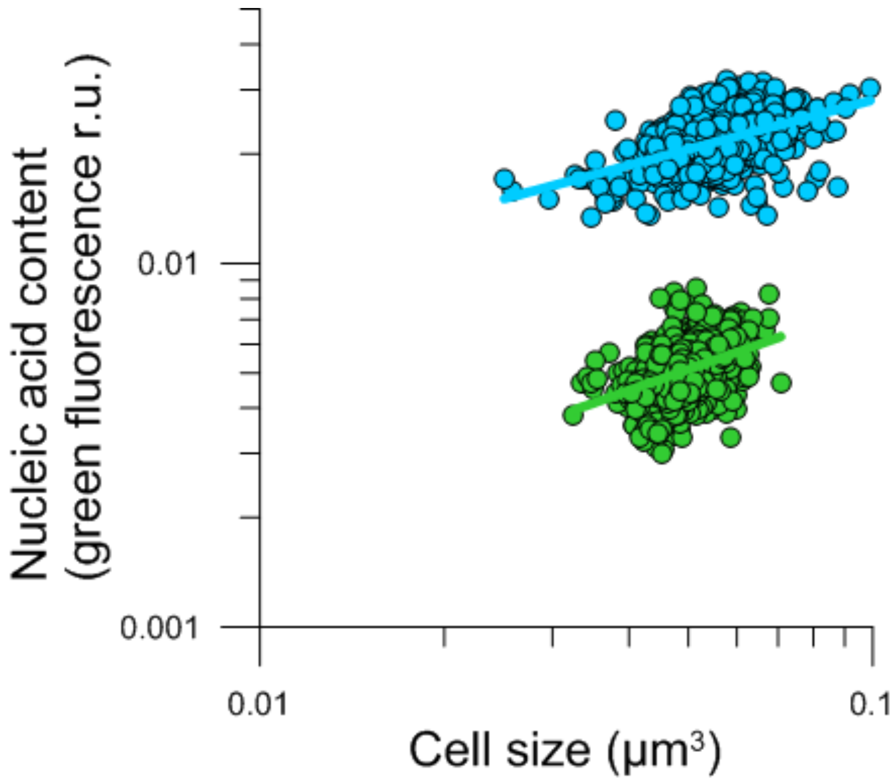

Fig. S3. Mean nucleic acid content as indicated by relative green fluorescence (FL1) after Syto-13 staining versus cell size (bv) of LNA and HNA bacterial cells of all samples collected in the study period (April 2002-March 2012). Details are given in. Calvo-Díaz & Morán (2006). Results agree with universal relationships between cell and genome sizes (Hessen *et al.*, 2013). Fitted lines represent ordinary least squares linear regressions: HNA $FL1_{HNA} = 0.002 + 0.062\ bv_{HNA}$, r^2=0.16, p<0.001, n=891, $FL1_{HNA} = 0.012 + 0.172\ bv_{HNA}$, r^2=0.23, p<0.001, n=884.

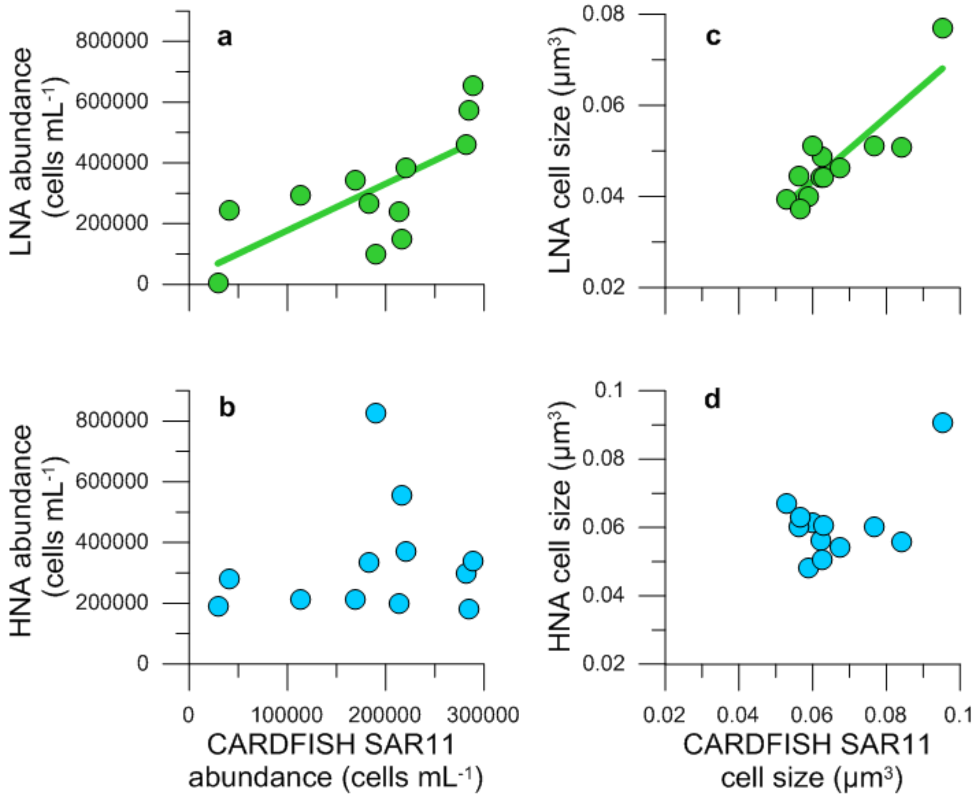

Figure S4. Relationships between LNA and SAR11 cells. Relationships between the abundance of LNA (a) and HNA (b) cells determined by flow cytometry and the abundance of SAR11 bacteria determined by CARDFISH in surface samples taken in 2012. Fitted line in (a): LNA = 24264 + 1.54 SAR11, r^2=0.50, p=0.01, n=12. Relationship between the cell size of LNA (c) and HNA (d) cells determined by flow cytometry and that of SAR11 bacteria determined by CARDFISH image analysis in surface samples taken in 2012. Fitted line in (c): LNA = 0.001 + 0.70 SAR11, r^2=0.74, p<0.001, n=12.

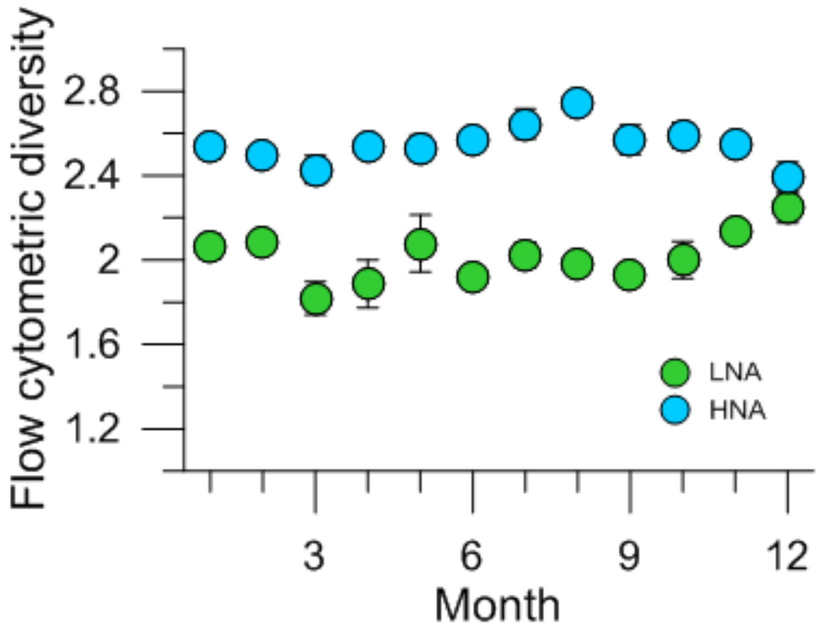

Fig. S5. Mean monthly values (± standard errors) of flow cytometric Shannon_Weaver diversity index of HNA and LNA bacteria in the upper mixed layer of the study site. Flow cytometric diversity was calculated as described in detail in García et al. (submitted), who also show that the seasonal patterns of flow cytometric and genetic diversity were in good agreement.

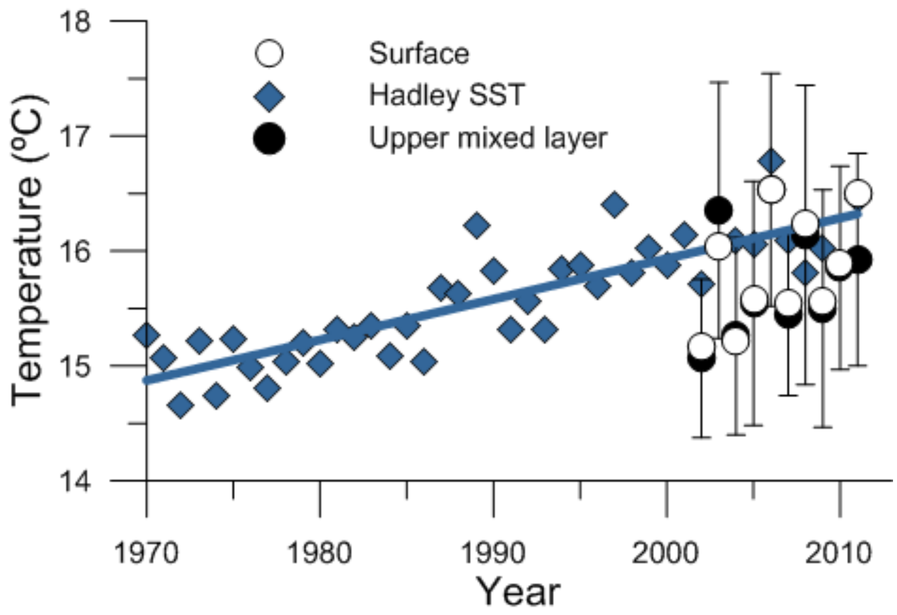

Fig. S6. Mean annual (April-March) Hadley sea surface temperature (SST) values from 1970 to 2011 corresponding to a 1° latitude-longitude pixel centred at 43.5ºN, 5.5ºE compared with mean annual temperatures at the surface and the upper mixed layer (± standard errors) of the study site. Hadley SST was significantly correlated with time ($r=0.84$, $p<<0.001$, $n=42$). Hadley SST data were obtained from http://www.metoffice.gov.uk/hadobs/hadisst/ (Rayner *et al.*, 2003).

References

Calvo-Díaz A, Morán XAG (2006) Seasonal dynamics of picoplankton in shelf waters of the southern Bay of Biscay. *Aquatic Microbial Ecology,* **42**, 159-174.

García F.C., Alonso-Sáez L., Morán X.A.G., López-Urrutia, Á. (submitted) Seasonality in molecular and cytometric diversity of marine bacterioplankton: the reshuffling of bacterial taxa by vertical mixing. *Environmental Microbiology*

Hessen DO, Daufresne M, Leinaas HP (2013) Temperature-size relations from the cellular-genomic perspective. *Biological Reviews,* **88**, 476-489.

Rayner NA, Parker DE, Horton EB *et al.* (2003) Global analyses of sea surface temperature, sea ice, and night marine air temperature since the late nineteenth century. *Journal of Geophysical Research-Atmospheres,* **108**.
